# Supplementary material for: Comparison of virtual reality and real environment effects on perception of height in healthy individuals
Source: PLoS One. 2025 Aug 26;20(8):e0330443. doi: 10.1371/journal.pone.0330443 (PMC12380285; doi:10.1371/journal.pone.0330443)
Supplement: Attachment — Submitted filename: Responds to reviewers comments.docx [file pone.0330443.s002.docx]

Jan 12^th^, 2025

**Mrs. Jeyasakthy Saniasiaya,**

**Academic Editor**

**PLOS ONE**

Dear Mrs. Jeyasakthy Saniasiaya,

According to your last email, please find attached a copy of the revised version of our paper, as a possible publication in PLOS ONE:

**“THE ROLE OF SACCULE IN HEIGHT PERCEPTION”** The comments and suggestions for revision are reflected in the current version of the paper.

A response letter with main changes has been done in this revision together a separated individual response for each reviewer to their comments and a specific *reference section* to support our answers are attached.

Looking forward to hearing from you.

Thank you.

Yours sincerely,

**Response letter to the Reviewers’ Comments on PLOS ONE**

**Title of the paper**: **The Role of Saccule in Height Perception**

First of all, authors would like to thank all the anonymous Reviewers and the Editors again for their efforts and valuable time to review and improve our paper.

Taking into account their constructive suggestions and comments, the paper has been carefully revised following the referees’ comments.

The main changes in the revised manuscript are:

1. Grammar and language organization
2. Introduction to the study
3. Materials and methods of the study

We provide below the responses to each referee’s comments.

**Editor Comments**

1. **Major grammatical and language editing required**

**The manuscript edited by an native speaker after artificial intelligence check.**

2. **Hypothesis of this study needs to be justified**

**The introduction was rewritten to clarify the hypothesis and the objectives of the study.**

3. **Methodology should be more detailed**

**Edits have been made in the material and method section.**

**Reviewer #1**

**Reviewer’s Comment 1:** Title: Consider changing title of the paper – as the given title is not adhere with the aim of the study.
‘Comparison of virtual reality and real environment effects on perception of height in healthy individuals’

The title of the article has been edited according to your suggestion. “Comparison of virtual reality and real environment effects on perception of height in healthy individuals’

**Reviewer’s Comment 2:** Page 4 Line 78: what is meant by high perception??

The stated section aims to define the perception of height.

In addition, in the aforementioned study, when estimating the size of objects, individuals may see objects smaller or larger than they actually are due to fear of height. Fear of heights can lead to distortion of individuals' perception when looking from a high place. This psychological effect also changes size estimations by affecting the perception of height.

**Reviewer’s Comment 3:** Page 5 Line 104-106: Rephrase the sentence for easy understanding.

In line with your suggestion, the specified part has been rearranged as “This study included participants between the ages of 20 and 50 with normal hearing, as confirmed by pure tone audiometry, Type A tympanograms, and acoustic reflexes. Participants also exhibited positive cVEMP results, no defined balance or visual impairment, and no fear of heights.”

**Reviewer’s Comment 4:** Page 5: Give a sub heading for designing the simulation of virtual reality environment.

In line with your suggestion, a sub-heading has been added to the material and method section. These sections were: a. VR environment creation, b. Preparation for the experiment:, c. Experiment implementation: d. Statistical analysis:

**Reviewer’s Comment 5** Page 6: Explain the audiological procedures, cVEMP and MMSE test procedures in detail In general, the SOT test gives information about the data use of patients in pathologies. This is in line with our work objective. In addition, it was evaluated that the other tests mentioned would not be reliable due to the fatigue frequently observed in MS patients.

Test procedures were explained in detail in the test preparation section of the material and methods chapter.

**Reviewer’s Comment 6:** Page 6: Give a sub heading for VR simulation environment and real environment.

The criteria and specifications for the VR test environment are specified in the “VR environment creation” section.

**Reviewer’s Comment 7:** Page 7 Line 146: the point at which people started to estimate the height was also changed – how it is changed, explain this in detail..

All the procedure rephrased as “During the experiment, participants were asked to estimate height. This estimation was designed to take place when looking down from above and when looking up from below. Here, in order to prevent the aforementioned exocentric mechanisms from being activated, care was taken to ensure that there was no floor number in the elevator and that they could not calculate the number of floors as mentioned in the virtual reality environment. In the real environment, participants were blindfolded and their eyes were not opened until they reached the edge of the window to avoid seeing floor numbers in the corridors, including the elevator. In addition to this, to eliminate the learning effect, half of the participants were randomly asked to make height estimations by first looking up from the bottom at preset heights and then looking down from the top by going up with the help of an elevator. The other half of the participants were first taken up by elevator and asked to estimate the height by looking from top to bottom. They were then taken down by elevator and asked to estimate the height by looking from bottom to top. This procedure was repeated in the virtual reality and real environment conditions. (Figure 1, Figure 2)”

**Reviewer’s Comment 8:** Page 8 Line 177-180: What is meant by current height? What is the defined value for the current height and how you defined.

We want the define current height by 24 meter which is defined in materials and methods section as “The ground clearance of the 5th floor was measured using a Bosch plr 25 laser measure (Germany) and found to be 24 metres.”

**Reviewer’s Comment 9:** What is the height of the individuals who had participated in this study, and how it influences the height perception; Provide the appropriate information in discussion section with references. Also, every individual’s height will be different and how it is analysed statistically.

Within the scope of the study, the distances between the people and the place they look at change as height plus height subtracted from height due to the position of the eyes in the upward gaze and the distance looked at as height plus height due to the position of the eyes in the downward gaze. In order to eliminate this effect, the calculations given in Table 2 were activated to exclude the neck effect on distance changes

The height of the participants was measured with a tape measure at the highest point of the head in an upright position against a wall. This part added the material and methods section.

**Reviewer’s Comment 10:** Table 3: why only 26 participants were included, but in methods section it is mentioned that the study included 52 participants.

When estimating height in the study, half of the participants looked down from above and the other half primarily looked up from below. Therefore, Table 3 includes 26 participants whose starting point was the ground floor (look up from below) and 26 participants whose starting point was the 5th floor(look down from above), for a total of 52 participants.

**Reviewer’s Comment 11:** Why Mean and SD values mentioned with comma???
Page 12 Line 223: evaluated the effect of this, ‘this’ means???

It was found that the mean and standard deviation values were written with commas in some of the tables and with periods in others, and this inconsistency was corrected and all tables were arranged in accordance with the journal's spelling rules.

The section has rephrased as “The aforementioned studies have shown that saccule functions are affected by changes in visual perception. However, the underlying cause of these effects is unknown. In our study, unlike these studies, we created an environment that alters visual perception but does not mechanically stimulate the saccule and evaluated its effect on height perception.”

**Reviewer’s Comment 12:** Figure 3-5: y-axis of the bar graphs denotes???

Necessary adjustments were made to the figures to determine the quantities. In Figures 3, 4 and 5, the y-axes represent the elevation (in meters).

**Reviewer’s Comment 13:** Figure 5: why y axis values are in 5 digits???

Necessary adjustments were made to the figures to determine the quantities.

**Reviewer’s Comment 14:** General: Maintain the uniformity in mentioning the term ‘virtual reality’ or simply use the abbreviated term ‘VR’

Necessary arrangements have been made

**Reviewer’s Comment 15:** General: Provide the expansion for the abbreviated terms used in the document for the first time.

Necessary arrangements have been made

**Reviewer #2**

This paper claims to look at the effect of mechanical stimulation of the saccule on height perception. However it is not very clear what is meany by mechanical stimulation. It seems to just mean going up in a lift, but the rationale as to why that should affect height perception is not given. Clearly the mechanical stimulation is not simultaneous with the height measurement, which is a limitation of the approach.

Saccule stimulation is mainly based on the activation of hairy cells as a result of mechanical impact. Height perception is basically based on the opinions of individuals. However, it has been investigated whether the saccule, which plays a critical role especially in the perception of upward movements, has an additional effect on the perception of these movements. In this context, we focused on the question of whether the perception of upward movements produces a change in the perception of the person depending on the movement, other than just simultaneous perception. In this context, instead of simultaneous measurements, we aimed to evaluate the effect of fluid movement in hairy cells caused by a mechanical effect and the changes created by this movement in hairy cells on height perception.

It is not clear to me that people would in general be reliable at height perception. That seems to be quite key to the experiment. There needs to be some evidence given that people are reliable at height perception, as if they are not, then the experiment is not likely to find anything significant. I would like to see data as to how reliable people are at height perception – ideally for some different heights. The choice of the 5th floor seems fairly arbitrary. Presumably at some point (say 100th floor), perception of height becomes very unreliable, but it is not clear at what point that would be the case, or whether we expect height perception at the 5th floor to be reliable.

This study is based on the assumption that people's height perception is reliable. Although we could not find a direct study on this issue, observations made during the research suggest that participants' perceptions of height and distance are often based on similar strategies (e.g., estimation based on a known distance). It was observed that participants frequently used egocentric and exocentric evaluation mechanisms, especially when estimating the height of apartments.

In egocentric evaluations, participants made estimates with reference to their height (e.g., “If I am 1.75 m tall, that is about 4 times as tall, i.e. 7 meters”). In exocentric evaluations, estimates were made using environmental elements (e.g., "If the height of this apartment is 3 meters, the 5th floor should be approximately 25 meters"). These estimates are based on the evaluation of visual data.

However, in our study, we aimed to investigate the role of the vestibular system (especially saccular stimuli) in height perception rather than the effects of 3D vision provided by the two eyes, taking necessary precautions to exclude the effect of visual data-based estimates. The results obtained from our study support the assumption that height perception is reliable, as the participants obtained similar results in top-down and bottom-up views.

The background is generally not well written. Many sentences need work and do not entirely make sense. In general the English in the paper is not very good, which is somewhat frustrating when trying to review the work. I don’t think it is my role as a reviewer to correct English. The manuscript should be better proof-read before submission to a journal.

We rewrite the introduction for to clarify the hypothesis and the aims of the study. The manuscript edited by an native speaker after artificial intelligence check.

The hypothesis of the experiment is not well defined. Are you assuming that the lift is stimulating the saccule and that this will somehow affect height perception? Why? Have others shown that (and if so what did they do in their experiments?). It seems quite tenuous. The points about correcting for the height of the subjects are not explained clearly/justified. There is no hypothesis stated, or any rationale given for the extra analysis.

Basically, there are no studies on how our perception of height is formed. In the existing literature, the use of visual data and the role of individuals' cognitive skills in environmental perception are emphasized. However, studies have shown that functional disorders of the saccule have an effect on cognitive skills and can therefore change environmental perception. In this context, in our study, we aimed to evaluate whether there is a perceptual difference between the conditions in which the saccule is stimulated by perilymphatic movement and the conditions in which perilymphatic movement is absent (i.e., not stimulated). Based on your suggestions, edits have been made to the introduction to express this purpose more clearly.

I am not convinced that going 5 floors in a lift is stimulating the saccule to a degree that we would expect a change in height perception (if we are assuming that there is evidence for saccular stimulation affecting height perception – although that is not clear from the manuscript). Also the non-simultaneous measurement of height with the life stimulation means that any affect on height perception could have recovered anyway. I therefore don’t think that the conclusion ‘Mechanical stimulation of the saccule is thought to have a limited role in height perception’ is justified from the work carried out.’

Since there is no previous study in this area, the 5th floor was chosen as the experimental environment. This floor is considered to be both easily accessible and a level where height perception may come to the fore in the visual perception of the participants, rather than floor estimation based on a more horizontal view, such as the first floor. In the first trials, it was observed that the participants calculated the height on the 1st, 2nd, and 3rd floors based on their horizontal viewing angles and the duration of their stay in the elevator. However, it has been found that this does not occur at higher floors. For this reason, the 5th floor was preferred as a level where the existing facilities and fear of heights would be minimal in the participants.

Given these limitations I don’t think that the work is suitable for publication at this time. Possibly the work comparing VR height perception to real-world height perception would be of interest to some in the VR community.

**Reviewer’s Comment 1:** : Abstract You probably don’t need to put the date.
It might help to say briefly what the mechanical stimulation is in the abstract.
Acoustic immittance (not ‘immitansmetry’)

In the abstract section, date information is omitted and mechanical stimulation is described in detail. Inconsistencies in the terms expressing clinical measurement were identified and the necessary arrangements were made and the expressions were revised to ensure terminological consistency.

**Reviewer’s Comment 2:** L53 I am not sure about your definition of balance

This definition was made by “Charles M. Plishka” in the text book dated “2015”.

**Reviewer’s Comment 3:** L54 which image?

Image defines “It refers to the visual perception of objects around the individual. The balance system allows the eyes to see the environment steadily during head movements, ensuring that the image remains clear and continuous.”

**Reviewer’s Comment 4:** L64/65 I don’t understand why visual perception should alter saccular function. Can you explain more?

Visual perception may affect saccular function because the vestibular and visual systems are integrated in processes such as spatial awareness and posture control. Visual cues may contribute to the reorganization of these processes by modulating the linear acceleration and gravity signals perceived by the saccule. This interaction becomes more evident especially in environments such as virtual reality applications where visual stimuli are intense.

Öztürk et al. showed that visual stimuli affect VEMP responses due to the vection effect. “Vection” is the phenomenon where a false self-motion sensation is created with visual stimuli covering the visual field of a person. Some of the side effects of this sensation can be nausea, imbalance, blurred vision, and headache in individuals”. Similarly, the study by Dündar et al. showed that upward and downward accelerations in an open-sided elevator caused increases in cVEMP responses.

The main mechanism of these effects is thought to be the "rewighting" mechanisms related to the balance-maintaining principle of the vestibular system. This mechanism refers to the adaptability of the system in processing sensory information and plays an important role in explaining the effects of visual-vestibular interactions on perception and balance.

**Reviewer’s Comment 5:** L69 can you be more specific what the link is between balance and cognitive skills

Balance and cognitive skills are strongly interconnected through common systems and processes in the brain. Cognitive functions such as attention, spatial awareness and sensory integration need to work effectively to maintain balance. Aging and neurological diseases can negatively affect these two areas, increasing the risk of falls. In addition, balance exercises can improve cognitive functions, and cognitive training can improve balance, revealing that these two systems work in harmony with each other.

Recently, the number of studies in this field has been increasing. The main studies that provide important findings are given below. However, due to reference limitations, the details of these studies could not be covered comprehensively.

Zwergal A, Grabova D, Schöberl F. Vestibular contribution to spatial orientation and navigation. Curr Opin Neurol. 2024 Feb 1;37(1):52-58. doi: 10.1097/WCO.0000000000001230. Epub 2023 Nov 27. PMID: 38010039; PMCID: PMC10779452.

Dieterich M, Brandt T. Central vestibular networking for sensorimotor control, cognition, and emotion. Curr Opin Neurol. 2024 Feb 1;37(1):74-82. doi: 10.1097/WCO.0000000000001233. Epub 2023 Nov 30. PMID: 38032266; PMCID: PMC10779454.

Zanchi S, Cuturi LF, Sandini G, Gori M, Ferrè ER. Vestibular contribution to spatial encoding. Eur J Neurosci. 2023 Nov;58(9):4034-4042. doi: 10.1111/ejn.16146. Epub 2023 Sep 9. PMID: 37688501.

Pfeiffer C, Serino A, Blanke O. The vestibular system: a spatial reference for bodily self-consciousness. Front Integr Neurosci. 2014 Apr 17;8:31. doi: 10.3389/fnint.2014.00031. PMID: 24860446; PMCID: PMC4028995.

**Reviewer’s Comment 6:** L69 poor sentence ‘it is stated that the balance system can be affected by the age of a person, his/her disorders, etc.’

In line with your suggestion, the specified sentence has been corrected as “It is important to know that the balance system's functionality can be significantly affected by age, current health status, and neurological or vestibular disorders..”

**Reviewer’s Comment 7:** L72 poor sentence ‘Studies have shown that VEMP results were the most effected test batteries by abnormal when compared with healthy individuals.’

In line with your suggestion, the sentence stated as “Research has demonstrated that VEMP tests are among the most sensitive and most affected test batteries for the detection of abnormal conditions when compared to healthy subjects. This underscores the significance of VEMP as a crucial instrument in the evaluation of functional disorders, particularly in the vestibular system.”

**Reviewer’s Comment 8:** L73-75 can you reference the evidence for this and explain conceptually why this should occur?

Hippocampal atrophy, the volumetric reduction of the hippocampus, is particularly evident in Alzheimer's disease and other types of dementia. The hippocampus is one of the earliest degenerating regions in the brains of Alzheimer's patients [1]. This region is considered to be one of the important centers of the spatial orientation network and, accordingly, balance [2]. Spatial disorientation, one of the earliest symptoms of Alzheimer's disease, is thought to be due to damage to this region.

It is thought that 4 main pathways are related to cognition in the vestibular system, which transmits information to the central nervous system.

(1) Vestibulo-thalamo-cortical pathway;
(2) A pathway from the dorsal tegmental nucleus to the entorhinal cortex through the lateral mammillary nucleus and the anterodorsal nucleus of the thalamus
(3) A pathway to the hippocampus through the nucleus reticularis pontis oralis, supramammillary nucleus and medial septum
(4) A possible pathway through the ventral lateral nucleus of the cerebellum and thalamus [3]

Hypotheses on the main reasons why the vestibular system affects cognitive activities:

Cognitive load hypothesis:Due to the allocation of limited resources to balance control, the amount of resources available for cognitive functions decreases.

Deprivation hypothesis: Reduced vestibular input causes accelerated brain atrophy, which leads to dementia. Hippocampal atrophy supports this hypothesis.

Social isolation hypothesis: Basically, the limitations experienced due to equilibrium problems socially constraining people.

Common causes hypothesis: It states that both conditions are actually caused by the same reasons and that these reasons may be occur together in people.

1. Braak H, Braak E. 1991. Neuropathological stageing of Alzheimer-related changes. Acta Neuropathol., 82, pp. 239-259.
2. Maguire EA, Frackowiak RS, Frith CD. 1997. Recalling routes around London: activation of the right hippocampus in taxi drivers. J. Neurosci., 17, pp. 7103-7110.
3. Hitier, M., Besnard, S., & Smith, P. F. (2014). Vestibular pathways involved in cognition. Frontiers in integrative neuroscience, 8, 59.

**Reviewer’s Comment 9:** L76 what is the link between cognitive skills and VEMP? Can you explain this and reference it?

Bosmans J, Jorissen C, Gilles A, Mertens G, Engelborghs S, Cras P, Van Ombergen A, Van Rompaey V. Vestibular Function in Older Adults With Cognitive Impairment: A Systematic Review. Ear Hear. 2021 Sep/Oct;42(5):1119-1126. doi: 10.1097/AUD.0000000000001040. PMID: 33974775.

In the conclusion section “Measurements of the VCR, as evoked by the VEMP test, discriminate between patients with cognitive impairment (MCI and AD) and older adults with preserved cognition, whereas measurements of the vestibulo-ocular reflex do not. More studies are needed to further elaborate on these findings.”

**Reviewer’s Comment 10:** L78 High not heigh?

High perception it is corrected in the height perception as.

**Reviewer’s Comment 11:** L82-84 Sentence needs work
Can you explain conceptually why the saccule should affect height perception?

In general, the processing of perceptions and, accordingly, the perception of our environment is recognised as a cognitive skill. The task of the vestibular system, especially the saccule, is the perception of upward and downward movement. The saccule is also known to be associated with cognitive disorders. In this context, it is thought that stimulation of the saccule may play a role in our perceptions and especially in our height perception due to cognitive effects.

**Reviewer’s Comment 12:**106-108 Did you exclude subjects from the study?

In general, we exclude only 1 participant because of the abnormal guess for height (200 metres). This value was so far from expectations that we excluded this competitor.

**Reviewer’s Comment 13:** 128-131 I am not convinced that subjects would be accurate in height estimation from a 5th floor in general. Do you have any evidence that subjects are reliable in doing this?

This study is based on the assumption that people's height perception is reliable. Although we could not find a direct study on this topic, observations made during the research suggest that participants' perceptions of height and distance were generally based on similar strategies (e.g., estimation based on a known distance). It was observed that participants frequently used egocentric and exocentric evaluation mechanisms, especially when estimating the height of apartments.

In egocentric evaluations, participants made estimates with reference to their height (e.g., “If I am 1.75 m tall, that is about 4 times as tall, i.e. 7 meters”). In exocentric evaluations, estimates were made using environmental elements (e.g., “If the height of this apartment is 3 meters, the 5th floor should be about 25 meters”). The basis of these predictions is the evaluation of visual data.

However, in our study, we aimed to investigate the role of the vestibular system (especially saccular stimuli) in height perception rather than the effects of 3D vision provided by the two eyes, taking the necessary precautions to exclude the influence of visual data-based estimations. The results obtained from our study support the assumption that height perception is reliable, as the participants obtained similar results in top-down and bottom-up views.

**Reviewer’s Comment 14:** P173 why is Wilcoxen in “”? Do you mean the Wilcoxen test?

The "" sign is used for emphasis and the Wilcoxon Signed Rank Test is what is meant here. In order to clarify this situation, necessary corrections have been made and the wording has been made clearer.

**Reviewer’s Comment 15:** P9 I don’t really understand the point of the analysis here. Won’t the VR and real world estimated include the height of the subjects?

**Reviewer’s Comment 16:** I don’t really follow the point of Table 3 either. Why are you using a Mann-Whitney test here when you used Wilcoxen before.

The height estimates obtained from the same participants in the virtual reality environment and in the real environment were compared and it was determined that the data did not conform the normal distribution. Therefore, Wilcoxon test was used to examine the difference between two dependent groups.

However, in Table 3, the height estimates made in the virtual reality environment and in the real environment are compared according to the height at which the estimates started. Since the groups were independent and it was determined that the data did not conform to a normal distribution, the non-parametric Mann-Whitney U test was preferred in this comparison.
