# Supplementary material for: Comparison of virtual reality and real environment effects on perception of height in healthy individuals
Source: PLoS One. 2025 Aug 26;20(8):e0330443. doi: 10.1371/journal.pone.0330443 (PMC12380285; doi:10.1371/journal.pone.0330443)
Supplement: Attachment — Submitted filename: Response to Reviewers 11.07.2025.docx [file pone.0330443.s003.docx]

July 10^th^, 2025

**Mrs. Jeyasakthy Saniasiaya,**

**Academic Editor**

**PLOS ONE**

Dear Mrs. Jeyasakthy Saniasiaya,

According to your last email, please find attached a copy of the revised version of our paper, as a possible publication in PLOS ONE:

**“COMPARISON OF VIRTUAL REALITY AND REAL ENVIRONMENT EFFECTS ON PERCEPTION OF HEIGHT IN HEALTHY INDIVIDUALS”** The comments and suggestions for revision are reflected in the current version of the paper.

A response letter with main changes has been done in this revision together a separated individual response for each reviewer to their comments and a specific *reference section* to support our answers are attached.

Looking forward to hearing from you.

Thank you.

Yours sincerely,

**Response letter to the Reviewers’ Comments on PLOS ONE**

**Title of the paper: Comparison of virtual reality and real environment effects on perception of height in healthy individuals**

First of all, authors would like to thank all the anonymous Reviewers and the Editors again for their efforts and valuable time to review and improve our paper.

Taking into account their constructive suggestions and comments, the paper has been carefully revised following the referees’ comments.

The main changes in the revised manuscript are:

1. Research question and hypothesis
2. cVEMP and saccular functions
3. Spatial cognition
4. Grammar check

We provide below the responses to each referee’s comments.

**Additional Editor Comments:** The authors need to focus on justifying the reserach question and the hypothesis clearly

Thank you for this valuable suggestion. We have revised the final paragraph of the Introduction to clearly articulate both the rationale and the hypothesis. Specifically, we state: *“In this study, we hypothesized that mechanical stimulation of the saccule—induced by vertical acceleration during elevator movement—may influence vertical distance perception in healthy individuals. Specifically, we aimed to investigate whether this effect differs between real and virtual environments.”*
Additionally, we elaborated on the physiological basis for this hypothesis and the relevance of investigating this phenomenon in healthy individuals with confirmed normal saccular function.

**Reviewer #3**

**Reviewer’s Comment:**

a. In my opinion, the claim that these paradigms can stimulate saccule function is not convincing. It is not clear to me how going up or down in a lift could generate linear accelerations that could mechanically stimulate the maculae (an issue that is not rigorously discussed in the text in my opinion).

We appreciate this important observation. The saccule is known to be responsive to linear vertical accelerations [1], even at low intensities, such as those generated during passive elevator movement. Prior studies have demonstrated modulations in vestibular-evoked myogenic potentials (cVEMP) in response to slow vertical motion or optical illusions simulating elevation [2] While elevator-induced accelerations are indeed subtle compared to active motion tasks, they are within the functional range of saccular detection, particularly for low-threshold mechanoreceptors [1]. Our elevator paradigm was designed to generate these subtle vertical accelerations without visual or auditory cues, thereby engaging the saccule through passive stimulation. This justification has been added and expanded in the Introduction section.

1. Curthoys I. S. (2020). The Anatomical and Physiological Basis of Clinical Tests of Otolith Function. A Tribute to Yoshio Uchino. *Frontiers in neurology*, *11*, 566895. <https://doi.org/10.3389/fneur.2020.566895>
2. Dündar D. (2022). Investigation of the effect of virtual reality environments on the vestibular system using the VEMP test. Thesis. Istanbul: Medipol University. <https://acikerisim.medipol.edu.tr/xmlui/bitstream/handle/20.500.12511/11963/Dundar-Dilara-2022.pdf?sequence=1&isAllowed=y>
b. On the other hand, the authors do not describe what kind of results they obtained in the previously performed cVEMP and oVEMP tests (what is considered a normal response by the authors; are there asymmetries in the inter-ear responses of the participants; what was the amplitude and latency of these responses; what was the amplitude and latency of these responses).

Thank you for pointing this out. All participants underwent cVEMP testing to confirm normal saccular function. Only those with clearly reproducible bilateral responses within normative amplitude (typically >100 µV) and latency ranges (p13 latency ~13 ms; n23 latency ~23 ms) were included. Any participant exhibiting absent, delayed, or asymmetrical responses was excluded from the study. A brief summary of these criteria and the screening outcome has been added to the Methods section.

c. I think you cannot extrapolate these conclusions without at least one control experiment with subjects with abnormal macular function (assessed by cVEMPs and oVEMPs), showing that in these cases height perception is significantly altered.

However, this study design was chosen for two main reasons: a. This study was specifically designed as a first step to investigate the perceptual consequences of mechanical and non-mechanical stimulation of the saccule in individuals with normal vestibular function. One of the main objectives was to establish a normative baseline in a population without balance issues and with normal saccule responses (assessed by cVEMP). These baseline data will serve as a reference for future studies involving participants with vestibular dysfunction. b. The examination of the effects of abnormal saccular function responses poses significant challenges due to the compensatory capabilities of the vestibular system and the functional principles of the saccule (unlike the semicircular canals, the saccule is divided into two parts by the striola without a counterpart on the opposite side).

d. There may be other conditions where spatial cognition is impaired (e.g. persistent perceptual postural motion sickness) where cVEMP and oVEMP tests are normal. Also the item discussing the relationship between cognitive dysfunction and vestibular impairment is also weak and needs to be better explored (I suggest

Harun, A., Oh, E. S., Bigelow, R. T., Studenski, S., and Agrawal, Y. (2016). Vestibular impairment in dementia. Otol. Neurotol. 37, 1137-1142. doi: 10.1097/ MAO.0000000000001157;

Smith, P. F. (2023). Aging of the vestibular system and its relationship to dementia. Curr. Opin. Neurol. 37, 83-87. doi: 10.1097/WCO.0000000000001231).

Thank you for this constructive suggestion. We have revised the Introduction to incorporate recent findings on the vestibular–cognition relationship. In particular, we now reference Harun et al. (2016), who reported vestibular deficits in patients with dementia, and Smith (2023), who highlighted age-related vestibular degeneration as a contributing factor to cognitive decline. These additions help clarify that vestibular inputs, including those from the otolith organs, are integrally involved in spatial awareness and cognitive functioning.

**Reviewer #4**

**Reviewer’s Comment:**

a. While the authors have improved on the previous reviewer’s comment and has written their hypothesis in more detail, the hypothesis is still implied rather than explicitly stated and can be improved.

In this study, we hypothesized that mechanical stimulation of the saccule—induced by vertical acceleration during elevator movement—may influence vertical distance perception in healthy individuals. Specifically, we aimed to investigate whether this effect differs between real and virtual environments

b. The manuscript has occasional grammatical errors and awkward phrasing and can be improved there (e.g., Participants were informed in writing with an informed consent form and their signed consent was obtained).

We have carefully revised the manuscript for grammatical correctness and clarity. The sentence mentioned by the reviewer has been corrected to:
*“All participants were provided with written informed consent forms, and written consent was obtained prior to participation.”*
We also reviewed the entire manuscript for similar issues and made necessary corrections to improve readability and scientific tone.

c. The authors need to elaborate on the rationale for using 5th floor in their study. The authors can cite prior literature or priori used here.

Thank you for pointing this out. The fifth floor (~24 meters above ground) was selected based on pilot testing that indicated participants could not use exocentric cues (e.g., counting floors) to estimate height in either the real or virtual environment. This level of elevation was also sufficient to engage height perception without inducing discomfort or height phobia. The simulation environment was specifically designed to eliminate such cues and provide a perceptually neutral platform. We have included this rationale in the revised Methods and Introduction sections, and where applicable, we cited relevant virtual height perception studies.
