# Supplementary material for: Comparison of virtual reality and real environment effects on perception of height in healthy individuals
Source: PLoS One. 2025 Aug 26;20(8):e0330443. doi: 10.1371/journal.pone.0330443 (PMC12380285; doi:10.1371/journal.pone.0330443)
Supplement: Attachment — Submitted filename: Reviewer comments.docx [file pone.0330443.s001.docx]

**Reviewer comments**

Title: Consider changing title of the paper – as the given title is not adhere with the aim of the study.

‘Comparison of virtual reality and real environment effects on perception of height in healthy individuals’

Page 4 Line 78: what is meant by high perception??

Page 5 Line 104-106: Rephrase the sentence for easy understanding

Page 5: Give a sub heading for designing the simulation of virtual reality environment

Page 6: Explain the audiological procedures, cVEMP and MMSE test procedures in detail

Page 6: Give a sub heading for VR simulation environment and real environment

Page 7 Line 146: the point at which people started to estimate the height was also changed – how it is changed, explain this in detail.

Page 8 Line 177-180:

What is meant by current height? What is the defined value for the current height and how you defined it???

What is the height of the individuals who had participated in this study, and how it influences the height perception; Provide the appropriate information in discussion section with references. Also, every individual’s height will be different and how it is analysed statistically.

Table 3:

why only 26 participants were included, but in methods section it is mentioned that the study included 52 participants.

Why Mean and SD values mentioned with comma???

Page 12 Line 223: evaluated the effect of this, ‘this’ means???

Figure 3-5: y-axis of the bar graphs denotes???

Figure 5: why y axis values are in 5 digits???

General: Maintain the uniformity in mentioning the term ‘virtual reality’ or simply use the abbreviated term ‘VR’

General: Provide the expansion for the abbreviated terms used in the document for the first time.
